# Evolutionary Analysis of International Scientific Output in Occupational Therapy from 1917 to 2020

**DOI:** 10.3390/ijerph182312740

**Published:** 2021-12-02

**Authors:** Maria Cristina Espinosa-Sempere, Virtudes Pérez-Jover, Jose A. Quesada, Adriana López-Pineda, Concepción Carratalá-Munuera

**Affiliations:** 1 Pathology and Surgery Department, Miguel Hernandez University, 03550 San Juan de Alicante, Spain; c.espinosa@umh.es; 2 Health Psychology Department, Miguel Hernandez University, 03202 Elche, Spain; v.perez@umh.es; 3 Clinical Medicine Department, Miguel Hernandez University, 03550 San Juan de Alicante, Spain; adriannalp@hotmail.com (A.L.-P.); maria.carratala@umh.es (C.C.-M.)

**Keywords:** bibliographic research, scientometrics, health occupations, MEDLINE

## Abstract

Published evidence on the progress of occupational therapy research from a broad perspective is limited. The purpose of this study was to analyze the international research productivity on occupational therapy from 1917 to 2020. This was a bibliometric study including articles indexed on MEDLINE, Scopus, and CINAHL. The literature search was conducted in June 2021 using the descriptor “occupational therapy” and the term “Ergotherap*”, and was limited to citable documents. Price’s law and Bradford’s law were applied to analyze a number of bibliometric indicators. Research on occupational therapy had an average annual growth rate of 26.4% and followed an exponential model. The top producing countries were the USA (21.52%) and the UK (6.07%). There is a high transience index of 74.81%. The top producing author was Kielhofner, G. (*n* = 132). Studies with the highest reported scientific evidence accounted for 1.13% (*n* = 638) of the total number of publications. More randomized controlled trials are necessary to increase the quality of the evidence base. Moreover, a greater collaboration between authors is needed for the professionalization of this research field.

## 1. Introduction

Scientific publication is the most objective and practical way of transmitting research results, and its visibility is a valued aspect for both researchers and research institutions and organizations [1]. The quantitative analysis of scientific publications through bibliometrics allows us to evaluate the results of scientific activity in terms of volume, visibility, evolution, and structure [2,3], and also helps us to know the relative weight of the different lines of research and methodological orientations [4,5]. The bibliometric studies can include quantity indicators, quality indicators, and structural indicators and they should be selected according to the study objective [6]. However, the quantity indicators (number of publications per year and author productivity index among other) are considered to be the most appropriate tool to measure the growth of knowledge [2,7]. The current interest in scientific performance as an indicator of quality and criterion to characterize the progress and development of a discipline has highlighted the importance of bibliometrics as a scientific field [3,8].

The growth of scientific production has strengthened the use of bibliometrics in occupational therapy, and the first bibliometric studies on different issues of this discipline appeared in the 1980s. Most of these studies analyzed the journals considered to be most representative of the subject area [9,10,11], while other authors studied a country or institution such as Brown et al. [12] who analyzed the research publications by Australian authors. Other researchers explored the scientific literature on a specific subject area of occupational therapy, for example, Larsson, Haglund, and Hagberg [13] focused on geriatrics, Gutman and Raphael-Greenfield [14] focused on mental health, and Castro-Alzate et al. [15] focused on mirror therapy. However, so far, fewer studies have analyzed occupational therapy research from a broad perspective. Among them, Brown et al. [16] analyzed diverse parameters of the scientific literature on occupational therapy indexed in the Science Citation Index Expanded (SCIE) or the Social Sciences Citation Index (SSCI) from 1991 to 2014. To the best of our knowledge, there is no bibliometric study exploring the evolution of the overall scientific literature on occupational therapy from its beginning until the present, and also integrating information from multiple databases.

Knowing the pathway of the research on occupational therapy helps to consolidate the scientific foundations on which this discipline is based on, and the future trends. Thus, the present study aimed to describe the international scientific productivity on occupational therapy from 1917 to 2020, indexed in MEDLINE, SCOPUS, and CINAHL databases, by analyzing the number of publications, the growth rate, the authors productivity index, the collaboration index, the geographical distribution, the distribution by journal, the topics, the document types, and the language.

## 2. Materials and Methods

This was a bibliometric study. The methodology was based on previous studies [17,18] and the bibliometric indicators were selected according to the study objective. Included documents were papers on occupational therapy, published from 1917 to 2020, and indexed in the National Library of Medicine’s MEDLINE, Scopus, and Cumulative Index of Nursing and Allied Literature Complete (CINAHL) databases.

The terms used in the search strategy included the descriptor “occupational therapy” and the free text word “Ergotherap*”. This term is used to denominate the discipline in countries such as France, Germany, and Canada. The search was performed covering the title and keywords fields and with no language restrictions in order to retrieve the maximum number of documents on occupational therapy. The literature search was conducted on 1 June 2021 by two authors collaboratively.

### 2.1. Search Strategies

In MEDLINE database: ((“occupational therapy”[MeSH Terms] OR (occupational therapies[Title] OR occupational therapist[Title] OR occupational therapists[Title] OR occupational therapy[Title] OR occupational therapy[Title])) OR (ergotherapeute[Title] OR ergotherapeutes[Title] OR ergotherapeutic[Title] OR ergotherapeutical[Title] OR ergotheraphy[Title] OR ergotherapic[Title] OR ergotherapie[Title] OR ergotherapiques[Title] OR ergotherapist[Title] OR ergotherapists[Title] OR ergotherapy[Title])) OR (ergotherapeute[Other Term] OR ergotherapeutes[Other Term] OR ergotherapie[Other Term] OR ergotherapy[Other Term]) AND ((Journal Article[ptyp] OR Meta-Analysis[ptyp] OR Review[ptyp] OR systematic[sb] OR Guideline[ptyp] OR Comparative Study[ptyp] OR Evaluation Studies[ptyp] OR Observational Study[ptyp] OR Validation Studies[ptyp] OR Multicenter Study[ptyp] OR Practice Guideline[ptyp] OR Clinical Study[ptyp]) AND (“1917/01/01”[PDAT]: “2020/12/31”[PDAT])).

In Scopus database: (title (occupational and therap*) or key (occupational and therap*) or title (ergotherap*) or key (ergotherap*)) and pubyear < 2021 and (limit-to (doctype, “ar”) or limit-to (doctype, “re”).

In CINAHL database: MM occupational therapy OR MH occupational therapy OR TI occupational therapy OR MH ergotherap* OR MM ergotherap* OR TI ergotherap*.

### 2.2. Inclusion Criteria

Citable documents according to the Institute for Scientific Information (ISI) definition (original research articles, review articles, proceedings papers, and technical notes) were eligible. Only research documents from 1 January 1917 to 31 December 2020 were included. Non-citable documents (editorials, discussions, meeting abstracts, book reviews, and news items) were excluded.

### 2.3. Study Variables

The authors reviewed each record retrieved and extracted the following data: (1) journal name, (2) year of publication, (3) authors’ surnames and initials, (4) number of authors, (5) name of first author, (6) institution or affiliation of the first author, (7) key words or Major MeSH, (8) country of publication, (9) language of publication, and (10) document type. The thematic was analyzed by means of descriptors or MesH, and categorized into “Sociodemographic” which included all descriptors referring to gender, age, country, and life cycle stage; “Research” which included descriptors referring to research methodologies, type of studies, and statistical tests; “Occupational Therapy specific” which included terms such as activities of daily living, occupational dysfunction, occupational exposure, occupational disease, or model of human occupational; and “Health Vocabulary” which included terms such as patient, sick, rehabilitation, disease, and fracture. Terms that were confusing or did not fit into the other categories were categorized as “Miscellany”.

### 2.4. Data Collection

Scientific research production was studied by analyzing the number of documents published per year for every author, along with the document type, country, institution, journal, and language. Global output of occupational therapy research was quantified by calculating the proportion of publications in the discipline relative to the total scientific research output available in each database. The growth in scientific literature was analyzed by applying Price’s law. According to Price’s Law [19], the normal form of growth of science is exponential and much faster than the growth of most social phenomena. This growth is such that every 10 to 15 years the existing information doubles with exponential growth, although this depends largely on the area of knowledge in question, since each discipline undergoes its own evolution, passing through various stages; a phase of exponential growth proper, in which the growth rate is proportional to the size of the sample, and a phase of linear growth, in which the growth rate is constant or independent of the size of the system. Moreover, we calculated the transience index and the productivity index of the authors [20] (PI, log of the number of articles per author), which enabled classification of authors according to four levels of productivity: occasional authors (PI = 0, only 1 publication); moderately productive authors (0 < PI < 1, 2 to 9 publications); highly productive authors (1 ≤ PI ≤ 1.3, 10 to 19 publications), and authors with maximum productivity (PI > 1.3, more than 19 publications).

We also analyzed scientific production according to co-authorship and geographical distribution. We used the collaboration index, i.e., the average number of authors per article, to measure the degree of collaboration between authors. Then, we drew a map of the scope of collaboration through an analysis of social networks by using the software tool VOSviewer v.1.6.11. We identified the 20 most productive authors and the patterns of co-authorship among them. The size of spheres of the map reflected the total number of recorded papers, and the thickness of the lines connecting two spheres reflected the number of articles published by two authors working in collaboration. The Scopus database was used to collect the authors’ information.

Regarding distribution by journal, we analyzed the information extracted from the ”periodical full” field, we examined the most productive journals and used Bradford’s law [21] to identify the most prominent journals in occupational therapy. Bradford’s law provides a mathematical model for analyzing dispersion, and states that if scientific journals on a given topic are arranged in decreasing order of productivity of articles, a core of journals more specifically devoted to the topic can be distinguished. The number of journals of each successive group, including the same number of articles on the topic as the core, will be proportional to 1:n:n2:n3. The graphic representation of the Bradford model consists of concentric areas (Bradford areas) arranged in decreasing order of productivity. Each zone contains a similar number of articles, but the number of journals increases as you move away from the core. To evaluate the visibility of the journals, the Journal Citation Reports (JCR) database on impact factors and the Scimago Journal Rank (SJR) were used.

Information on publication year, document type, and language and country of publication was obtained from the corresponding fields (PY, Reference Type, LA, and Author/Address, respectively). Information on the topic of the articles came from the KW (key words) field. All data were entered into the RefWorks 6.0 reference manager.

### 2.5. Statistical Analysis

Data from RefWorks were exported to Microsoft Excel and the text variables were normalized (removing blank spaces, commas, full stops, and hyphens). Then, the database was exported to SPSS software to perform the statistical analysis. Values were expressed as frequencies, percentages, and cumulative percentages. Data were analyzed by decades (1911–1920, 1921–1930, 1931–1940, 1941–1950, 1951–1960, 1961–1970, 1971–1980, 1981–1990, 1991–2000, 2001–2010, 2011–2020), differentiating between the decades of the 20th century (until 1991–2000) and those of the 21st century (2001–2020). To evaluate whether an increase in scientific publications followed Price’s law of exponential growth, linear and exponential adjustments were made on the data obtained. To determine the Bradford zones, a semilogarithmic diagram was created to represent the cumulative number of articles against the log of the cumulative number of journals. Once the data were represented, it is possible to discern that the quantity of articles is divided into several parts. This model enabled the identification of the journals publishing the most articles on occupational therapy.

## 3. Results

**Figure 1:**
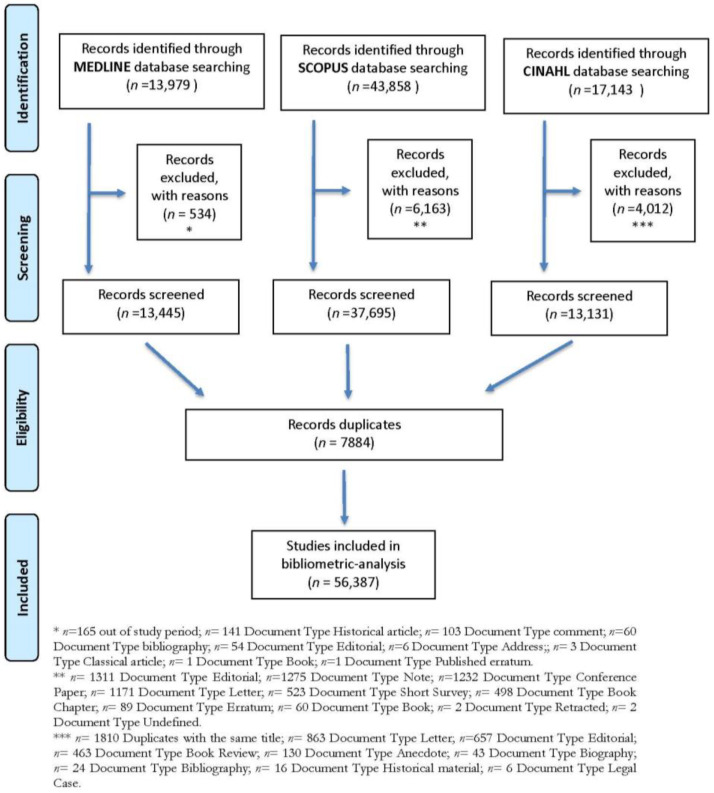
Study flow diagram.

The search strategy yielded a total of 56,387 records related to occupational therapy, published from 1917 to 2020 and indexed in MEDLINE, Scopus, and CINAHL (Figure 1). The mean annual contribution of occupational therapy research to global scientific production over the study period was 42.27 papers per 100,000 publications indexed in MEDLINE, 55.56 papers in SCOPUS, and 172.2 papers in CINAHL.

The growth rate (percentage change) of occupational therapy scientific literature since its inception is 254,200%. The growth rates per decade in the 20th century were 12,800% (1921–1930), 81.39% (1931–1940), 119.23% (1941–1950), 196.10% (1951–1960), 72.42% (1961–1970), 74.46% (1971–1980), 23.84% (1981–1990), and 32.16% (1991–2000). In the 21st century, the growth rate from 2001 to 2010 was 71.77% and in the decade 2011–2020 the literature grew by 49.32%. The mean annual growth over the study period was 26.4%. Our results confirm that the research on occupational therapy agrees with Price’s law, and the exponential model fit data properly (y = 5 × 10^−46^ e^0.0557x^, R^2^ = 0.9188) (Supplementary Materials Figure S1). In the study period, there were a total of 107,364,160 scientific publications indexed in the MEDLINE, SCOPUS, and CINAHL databases. Thus, the records relating to the field of occupational therapy represented 0.06% of the total scientific production.

The retrieved documents were produced by researchers in 121 countries. The most producing country is United States (USA) (21.52%, *n* = 12,133), followed by the United Kingdom (UK) (6.07%, *n* = 3423), Canada (5.56%, *n* = 3134), and Australia (5.55% *n* = 3129). Consistent with the countries where the documents were produced, English was the predominant language of publication (85.40%, *n* = 48,159). A smaller proportion was published in German (5.43%, *n* = 3061), French (1.91%, *n* = 1080), Russian (1.60%, *n* = 901), and Spanish (0.72%, *n* = 405). The representation of Latin American countries was 1.60% (*n* = 904).

**Figure 2:**
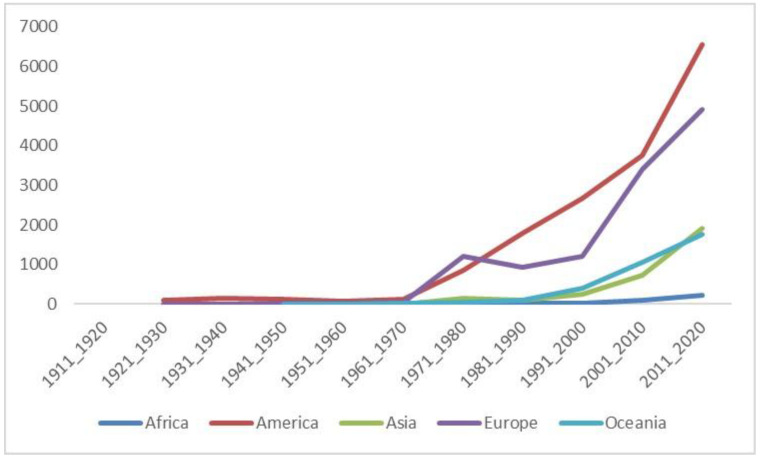
Scientific production on occupational therapy by geographical region and decades.

Figure 2 shows the proportion of scientific output by geographical region and periods of years. Scientific production was low during the first years of the study. The Americas was the most productive region in all decades except in the 1970s, which was Europe. The scientific production of the European continent grew irregularly in the 20th century. From the 2000s onwards, there was a remarkable increase in scientific production in all continents, with the Asian continent registering the highest increase in scientific production (166.99%) in the decade 2011–2020, even surpassing Oceania. Supplementary Materials Table S1 shows the most productive countries in the study period per continent, and their scientific production per decade. On the American continent, the USA topped the list of countries by decade, followed by Canada and Brazil since 1991. On the European continent, the UK ranked first, except for the decades 1971–1980 and 1981–1990, when it was overtaken by Germany. The second position on the European continent was held by Germany, except in the decades 1991–2000 and 2011–2020 when it was held by Sweden. In Oceania, the largest producer was Australia, followed by New Zealand. On the Asian continent, Japan was the largest producer during the study period, followed by China, however, the analysis by decade showed variability among the top countries. On the African continent, the top producer was South Africa for all studied decades, followed by Egypt and Nigeria.

During the study period, the most common document type was the journal article (94.75%, *n* = 53,426), followed by case study (1.77%, *n* = 997), review (1.00%, *n* = 562), and systematic review (0.55%, *n* = 308). There were 764 documents (1.35%) corresponding to other research papers (comparative studies, observational studies, and guidelines). Together, the papers reporting the highest level of evidence (meta-analyses, systematic reviews, clinical trial, randomized controlled trial, and controlled clinical trial) numbered 638 (1.31%). Thirty-eight (5.96%) documents of these were meta-analyses (0.07% of all papers) and 292 (45.77%) papers were clinical trial type papers (0.52% of all papers). The first recorded paper reporting a level I of evidence was a controlled clinical trial published in 1967 in the American Journal of Occupational Therapy by Wyrick, J. M. and was entitled ”Lack of effect on attitude change of two films dealing with cerebral palsy”. Table 1 shows the number of papers reporting the highest level of evidence by decade.

All clinical trials were published by authors from a total of 29 countries. The USA tops the list with 73 documents (11.44%), followed by Australia (*n* = 55, 8.62%), Canada (*n* = 41, 6.43%), UK (*n* = 27, 4.23%), and Brazil (*n* = 24, 3.76%). The list of the countries is completed by Sweden, Denmark, Germany, Spain, Japan, Netherlands, China, India, New Zealand, Ireland, Switzerland, Israel, Italy, Norway, Austria, Afghanistan, Argentina, Belgium, Colombia, Deutschland, Iran, Malaysia, Poland, and South Korea.

We identified 65,533 unique authors, 49,028 of whom were occasional authors signing a single paper (transience index and PI = 74.81%). The rest of the authors were classified as follows: 24.11% (*n* = 15,803) were moderately productive; 0.80% (*n* = 525) were highly productive; and 0.27% (*n* = 177) were authors of maximum productivity. The top 20 producers over the study period are listed in Table 2. Supplementary Table S2 provides a summary description of the 20 most productive first authors. The signature that most frequently appeared in the first position of the byline was Strzelecki, M. V., with 70 contributions (0.12% of total publications in the subject area), followed by Brown, T. (*n* = 66, 0.12%) and Kielhofner, G. (*n* = 59, 0.10%). If we consider all authors, regardless of the position of their signature, Kielhofner, G. is also the most prolific author, with 132 publications (0.23% of all publications), followed by Brown, T. (*n* = 126, 0.22%), and Eklund, M. (*n* = 119, 0.21%). The author with the highest *h*-index was Law, M., at 62, followed by Gitlin, L., at 49, and Polatajako, H., at 44. No information was obtained on the affiliation and *h*-index of Strzelecki, M. V. and Oliveck, M.

Table 3 shows the most productive authors by decades of study and the percentage of production with respect to the total number of publications in the period. Several authors stand out as top producers in various decades such as Pollock, H. who does so in the decades 1921–1930 and 1931–1940. West, W. L. appears as the top producer in the decades 1951–1960 and 1961–1970; Rogers, J. C. does so in the decades 1971–1980 and 1981–1990; and finally, Kielhofner, G. stands out as a top producer in the decades 1981–1990 and 1991–2000. The table shows an upward trend in the number of publications per author and decade, although the percentage of published papers out of the total number of papers in the decade is similar in all decades.

Table 4 shows the top 20 producing institutions ranked by number of articles. Eight universities were in the USA, seven universities in Canada, five universities were in Australia, and two universities were in Sweden. The institution that published the greatest number of papers was the University of Queensland (*n* = 326), followed by the University of Sydney (*n* = 215), both in Australia. Third place was taken by the University of Toronto in Canada (*n* = 213). All the institutions correspond to universities.

**Figure 3:**
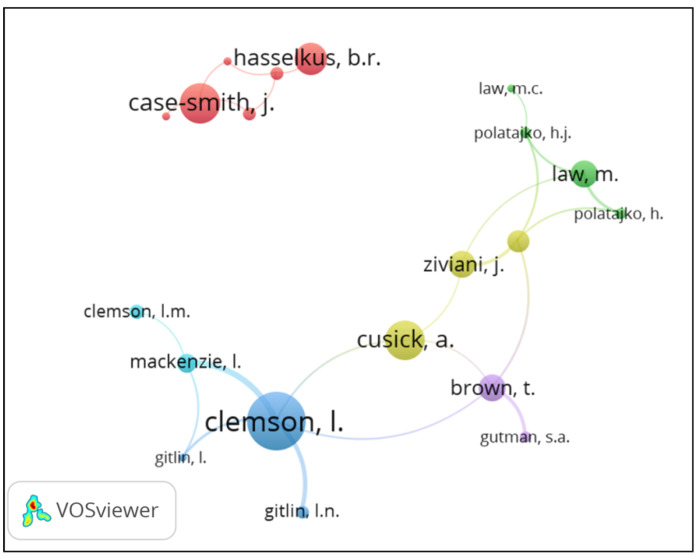
Graphic representation of a collaborative network between the major producing authors.

Regarding the co-authorship index (mean number of authors per publication), this increased from 1.0 in 1918 to 5.14 in 2020; it stood at 2.82 for the study period as a whole. The analysis of the co-authorship networks among the most productive authors revealed several research clusters. Figure 3 shows these collaborative networks among the most productive authors. The size of the cluster is proportional to the number of registered papers, and the size of the lines reflects the number of shared papers. The analysis shows co-authorship relationships between the major Australian producers. It reflects two Australia-Canada relationship with co-authorship between Rodger, S. and Polatajko, H., and between Ziviani, J. and Law, M. In addition, relationships between the USA and Australia were observed through Brown, T. and Gutman, S., and Clemson, L. and Gitlin, L. Although the most productive country is the USA, no strong research networks were identified among the most productive authors from this country. There were no differences found between the co-authorship rates of the top producing countries, i.e., 3.09 for the USA, 3.17 for UK, 3.49 for Australia, and 3.42 for Canada. Figure 3 shows a graphical depiction of this network.

The research topics were analyzed according to key words or MeSH descriptors; there were a total of 1,037,152 terms associated with the body of included documents. The highest number of terms identified per document was 723, but the most frequent quantity was 4. The three most common descriptors were “humans” (5.30%, *n* = 55,041), followed by “occupational therapy” (3.10%, *n* = 32,186), and “article” (2.10%, *n* = 21,753). The topics of the 100 most frequent descriptors (33.51%, *n*= 347,504 of the total) were analyzed and categorized into “sociodemographic” descriptors (40.43%, *n* = 140,500), “research”, for example, study types or study methodology (18.76%, *n* = 65,183), “occupational therapy specific” (16,86%, *n* = 58,600), “health vocabulary” (18,78%, *n* = 65,183), and “miscellany” (5.17%, *n* = 17,958). The most studied topic categories were activities of daily living (0.57%, *n* = 5966), occupational disease (0.47%, *n* = 4900), and occupational exposure (0.22%, *n* = 2338), all of these are within the “occupational therapy specific” category. The first pathology-related term to appear was stroke rehabilitation (0.13%, *n* = 1399), followed by mental disorder/rehabilitation (0.213%, *n* = 1352).

**Figure 4:**
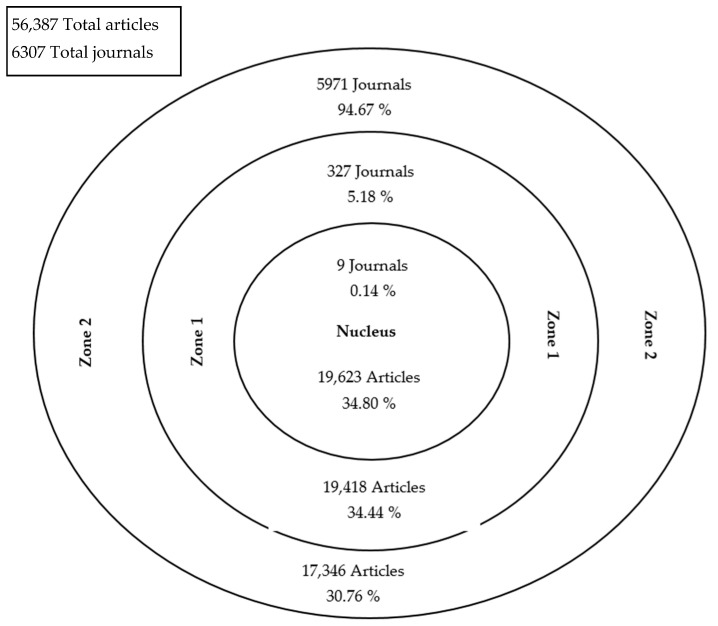
Diagram of Bradford distribution from 1917 to 2020.

The included documents in the present study were published in 6307 journals, 2882 of which published a single article in the subject area. Bradford’s distribution for occupational therapy from 1917 to 2020 is shown in Figure 4. The core of journals consists of 9 journals (0.14% of total number of journals) containing 19,623 documents (34.80% of total production in the study period). Table 5 presents the core journals publishing research in the field of occupational therapy, along with their main output and impact indicators. All core journals were indexed in JCR (Journal Citation Reports) except for *Occupational Therapy Now* which was indexed in the SJR (Scimago Journal Rank). All JCR-indexed journals were in the ”rehabilitation” category and *Occupational Therapy Now* was within the categories ”physical therapy, sports therapy and rehabilitation”, “public health, environmental and occupational health”, “rehabilitation”, “advanced and specialized nursing” of the SJR.

## 4. Discussion

The findings of the present study support the following implications regarding occupational therapy research: (1) More randomized controlled trials are necessary to increase the quality of the evidence base. (2) A greater collaboration between authors is needed for the professionalization of this research field, given that studies with multiple researchers have a higher impact and better quality than those produced by individuals. (3) It is desirable to use the descriptor ”occupational therapy” to index the studies in this discipline in order to avoid their classification as documents pertaining to other areas of knowledge like psychology.

### 4.1. Rate of Growth

This bibliometric study shows that occupational therapy research has grown considerably since its inception, although the field’s overall contribution to global scientific production is still modest. Scientific output increased exponentially, confirming Price’s growth model and situating the subject area as an emerging field. However, the growth rate decreased in the last decades which might be indicative of the beginning of discipline consolidation according to Price’s law, since a trend towards linear growth was observed. From the 1960s to 2000, the rate of growth decreased. The pace of publication began to quicken again in the 2000s, stimulated by diverse specialist organizations [22,23,24]. This trend may also be related to an increase in the number of occupational therapy journals, the globalization of scientific knowledge, and the improvement of electronic access to different databases. It is worthwhile to highlight the large increase in scientific literature from European continent during the decade 1971–1980. This strong growth may be due to the impulse given by the incorporation of European countries to the World Federation of Occupational Therapists in this decade (Finland in 1972, Austria in 1978, Italy in 1978, and Spain in 1978) and in the previous decade (Switzerland in 1962, France in 1964, Portugal in 1964, Belgium in 1968, and Ireland in 1970). The ensuing decline in European scientific production coincides with a decline in the annual growth rate of the economy for the European Union as a whole, and this could be a possible explanation for this evolution [25].

### 4.2. Authorship and Collaboration

With regard to the level of productivity, the high transience index indicates that this discipline is still young; the vast majority of authors make only isolated research contributions, and very few stable research groups exist [26]. The transience index is generally lower in more firmly established disciplines such as medicine. One explanation for the relatively low productivity is the concentration on clinical care in the discipline, as researchers may devote more time to their role as clinicians, to the detriment of research and dissemination activities [27,28]. Regarding the co-authorship index, the study results show scarce collaboration between the authors with the greatest scientific production. However, our data also show that collaboration has steadily increased, especially since 2010. A previous bibliometric study on occupational therapy publications by Australian authors reported a variation of the co-authorship rate from 1.3 in 1991 to 4.2 in 2015 [12]. This upward trend can be explained by the need for larger, more statistically powerful studies, the availability of funding to support cross-national data collection, and the availability and ease of use of social networks as a means to increase dialogue between research experts [29].

The most productive author of the study period was Kielhofner, G., affiliated with the University of Illinois in United States. It is striking that the most productive authors have made their contributions in the last 30 years, with the exception of Kielhofner who started in the mid-1970s. This finding shows that there has been greater interest in research and publishing results in the late 20th and 21st century.

### 4.3. Countries of Publication

Regarding output by countries, the USA dominated research activity over the study period as a whole, publishing more than 20% of the research produced worldwide. The UK, Canada, and Australia followed at a much more modest level. The results of the bibliometric study by Brown et al. [16] ranked Australia (*n* = 673) in second position followed by Canada (*n* = 667) and the UK (*n* = 613). These differences in positions may be due to the fact that the present study included documents from three different databases, broadening the representativeness of European countries. Nevertheless, the countries highlighted as the largest producers are those at the origins of occupational therapy. The low representation of Latin American countries among producers is striking, as occupational therapy began to develop there in the late 1950s, and they have a large number of university occupational therapy programs [30]. This may be due to the fact that Latin American authors tend to publish in national journals [31], which are not indexed in the major databases consulted by researchers. A bibliometric study using Latindex- indexed journals would be of interest to know the characteristics of occupational therapy research in these countries. A study by Samimi and Roshan [32] determined a bidirectional relationship between scientific production and a country’s gross domestic product (GDP), i.e., an increase in one or the other could increase the other. The present study found that the countries with the lowest scientific production were developing or poorer countries (Senegal, Rwanda, Krivorozhye, Kazakhstan, and Dominic Republic).

### 4.4. Types of Document

In terms of the type of document, despite an increase in the number of publications, there is a need to further promote studies with a high level of evidence (randomized clinical trials, systematic reviews, and meta-analyses) to strengthen the evidence base for the professional practice of occupational therapy, as indicated by Andresen et al. [22]. Other studies have found that the proportion of papers with level I evidence was higher in the field of obstetrics (6.95%) [33] or in the field of physiotherapy (17.76%) [34] than in the fields of occupational therapy. Clinical trials are considered to be the most reliable study design for evaluating the efficacy and safety of health interventions, as they are subject to the lowest degree of bias [33,35]. The results shown by our research indicate a progressive increase in the number of studies of this type. There is indeed a clear interest in recent years in doing quality science in occupational therapy. It seems that the stimulus provided by different organizations to improve occupational therapy research [22,23,36] and the commitment to evidence-based occupational therapy practice, which implies more training in scientific skills [37], is bearing fruit.

### 4.5. Journals

A core of nine journals that account for about one-third of the scientific output of occupational therapy was identified. The most productive journals for occupational therapy were those founded before 1980s: the *American Journal of Occupational Therapy*, the *British Journal of Occupational Therapy*, the *Australian Occupational Therapy* journal, and the *Canadian Journal of Occupational Therapy*. These journals can be considered to be the foundational journals of the discipline. The results by Brown and Gutman [38] agreed with the key importance of these journals to the field. The *Australian Occupational Therapy Journal* ranks third, and while it was not in print during the early years of the study period, its development has been significant, as other bibliometric studies indicated [11,16]. Half of the documents analyzed in the present study were published in journals specializing in this discipline [9,11,16,22,39]. It is striking to find *OT Practice* and *Occupational Therapy Now* among the core journals, ranking in the fifth and ninth positions, respectively; despite starting to be published in 2006, they are located in the area of the most influential journals for the profession. These journals are published by the American Association of Occupational Therapy and the Canadian Association of Occupational Therapy, respectively, and both have an *h*-index of 8. The appropriate JCR category to identify journals on occupational therapy is ”rehabilitation” and the best SJR subject categories are “physical therapy, sports therapy and rehabilitation”, “public health, environmental and occupational health”, “rehabilitation”, “advanced and specialized nursing” within the subject area of “health professions”.

### 4.6. Study Limitations

A broad and exhaustive search using MEDLINE, SCOPUS, and CINAHL databases was performed to minimize the methodological limitations derived from the retrieval of records. This ensured the correct use of bibliometric indicators and minimized the relativity of the data [40]. The MEDLINE database is the most widely used by the international scientific community; SCOPUS is an international multidisciplinary database of bibliographic references and citations, which allows different search options and provides impact metrics such as the *h*-index or the SJR; and the CINAHL database is an essential tool to find nursing, physiotherapy, and occupational therapy research.

With regard to the search strategy, we consulted and validated the strategy with a documentalist. In those databases that allowed the use of a controlled vocabulary (MEDLINE), thesaurus-controlled language was used to favor the elimination of synonyms, reduce ambiguities, and, above all, for precision in the search language using the descriptors (MeSH). The SCOPUS and CINAHL databases were searched for the descriptor ”Occupational Therapy”. This search strategy was specifically designed to avoid possible false positives. However, we assumed that articles in which these descriptors were not used could not be retrieved.

The study findings should be interpreted taking into consideration the limitations of the bibliometric indicators [2]. It is important to avoid the use of just one indicator, without complementing it with other information and without attending to the characteristics of the discipline in which it is being applied [2,18]. The bibliometric indicators used in this study focused on the measurement of the quantity of scientific production and not on the quality of the published studies, with the advantage that these indicators are well established and allow comparisons with previous bibliometric studies. However, further studies are needed to analyze the quality indicators of the research on occupational therapy.

## 5. Conclusions

During the study period, the growth rate of scientific production in occupational therapy has been exponential with a mean annual growth rate of 26.4%. The retrieved records in occupational therapy represented 0.06% of global scientific production, and more than a half of the articles were published in the 21st century. The most frequent type of study was the journal article and clinical trial studies accounted for 0.52% of all recorded occupational therapy publications. The growth rate of clinical trials increased during the study period, especially during the 21st century (120.9%).

There is a high number of authors who have only published a single paper, indicating that this is a young and emerging discipline. The most productive authors for the entire study period were Kielhofner, G. affiliated with the University of Illinois in United States; Brown, T., affiliated with Monash University in Australia; and Eklund, M., affiliated with Lunds Universitet in Sweden. The highest-producing countries were the USA and the UK. The most important journal in quantitative terms was the *American Journal of Occupational Therapy*.

The specific occupational therapy topics most studied were “activities of daily living”, “occupational disease”, and “occupational exposure”. The most frequently studied pathologies were stroke and mental disorders.

## Figures and Tables

**Table 1:** Number of papers reporting the highest level of evidence by decade.

	Decade	Papers with Level of Evidence *n* (%)	Clinical Trials *n* (%)
20th century	1967–1970	7 (1.10)	7 (2.40)
	1971–1980	11 (1.72)	11 (3.77)
	1981–1990	35 (5.49)	33 (11.30)
	1991–2000	57 (8.93)	54 (18.49)
21th century	2001–2010	107 (16.77)	70 (23.97)
	2011–2020	421 (65.99)	117 (40.07)
	Total	638	292

**Table 2:** The top 20 producers over the study period.

Author	Affiliation	h-Index	Country	Total Documents	% Contribution ^a^
Kielhofner, G.	University of Illinois at Chicago	33	United States	132	0.23%
Brown, T.	Monash University/Faculty of Medicine/Associate Editor/School of Primary and Allied Health Care	26	Australia	126	0.22%
Eklund, M.	Lunds Universitet/Institutionen for Halsovetenskaper/Malmo Hogskola	36	Sweden/Denmark	119	0.21%
Rodger, S.	The University of Queensland/Cooperative Research Centre for Living with Autism CRC	34	Australia	110	0.20%
Law, M.	McMaster University/CAnChild Centre for Childhood Disability Research	62	Canada	103	0.18%
Lloyd, C.	UNSW Sydney/Black Dog Institute	23	Australia	92	0.16%
Ziviani, J.	The University of Queensland/Children’s Health Queensland	42	Australia	82	0.15%
Clemson, L. M.	The University of Sydney/Monash University/Hornsby Ku-Ring-Gai Hospital	34	Australia	79	0.14%
Polatajko, H. J.	University of Toronto/Evelina London Children´s Healthcare	44	Canada/United Kingdom	74	0.13%
Gutman, S. A.	Columbia University/Rutgers University-Newark Campus	14	United States	74	0.13%
Nelson, D. L.	The University of Toledo	21	United States	73	0.13%
Strzelecki, M. V.		1		71	0.13%
Gitlin, L.	Drexel University/Johns Hopkins Universityh	49	United States	70	0.12%
Fisher, A. G.	Colorado State University/Umea Universitet	34	United States/Sweden	70	0.12%
Strong, J.	Royal Brisbane and Women´s Hospital/The University of Queensland	34	Australia	68	0.12%
Cusick, A.	The University of Sydney/Western Sydney University	23	Australia	68	0.12%
Mackenzie, L.	The Univerdity of Sydney	21	Australia	68	0.12%
Fleming, J.	The University of Queensland	34	Australia	67	0.12%
Hinojosa, J.	NYU Steinhardt	16	United States	67	0.12%
Mckenna, K.	The University of Queensland	31	Australia	66	0.12%

^a^ Proportion of documents in which the author appears out of the total documents included in this study (*n* = 56,387).

**Table 3:** The most productive authors per decade. The number of documents published and the percentage of the total number of publications registered in the decade are shown.

	Documents Published by Decade (*n*, %)										
Author	1911–1920	1921–1930	1931–1940	1941–1950	1951–1960	1961–1970	1971–1980	1981–1990	1991–2000	2001–2010	2011–2020
Neall, M. A.	1 (100)										
Haas, L. J.		7 (5.42)									
Pollock, H. M.		4 (3.10)	11 (4.70)								
Carr, B. W.		3 (2.32)									
Mack, G. M.			8 (3.42)								
Slagle, E. C.			4 (1.71)								
Giden, F. M.				4 (0.78)							
Casson, N. E.				5 (0.97)							
Licht, S.				14 (2.73%)							
Shali, K. H.					9 (0.59)						
West, W. L.					10 (0.66)	14 (0.53)					
Ayres, A. J.					11 (0.72)						
Conte, W. R.						10 (0.38)					
Llorens, L. A.						15 (0.57)					
Rogers, J. C.							13 (0.28)	29 (0.49)			
Hightower-Vandamm, M. D.							17 (0.37)				
Johnson, J. A.							36 (0.79)				
Barris, R.								31 (0.53)			
Kielhofner, G.								42 (0.72)	33 (0.43)		
Law, M.									31 (0.4)		
Nelson, D. L.									32 (0.41)		
McKenna, K.										57 (0.43)	
Rodger, S.										65 (0.49)	
Strzekecklu, M. V.										70 (0.53)	
Eklund, M.											53 (0.27)
Waite, A.											58 (0.29)
Brown, T.											98 (0.49)

**Table 4:** The top 20 producing institutions ranked by number of articles.

Institution	N Documents	Country
University Of Queensland	326	Australia
University Of Sydney	215	Australia
University Of Toronto	213	Canada
McMaster University	191	Canada
University Of Southern California	143	EEUU
La Trobe University	138	Australia
University Of Illinois	128	United States
Lund University	124	Sweden
University Of British Columbia	120	Canada
McGill University	114	Canada
Karolinska Institutet	111	Sweden
University Of Alberta	110	Canada
Dalhousie University	91	Canada
University Of Washington	90	United States
Monash University	80	Australia
University Of Western Ontario	71	United States
University Of Pittsburgh	69	United States
University Of California	66	United States
University Of Newcastle	66	Australia
Queen´S University	64	Canada

**Table 5:** Characteristics of core journals for occupational therapy, 1917–2017.

Journal	N Docs	%	h-Index	Quartile ^a^	Country
Am J Occup Ther	7249	12.86	82	Q1	USA
Br J Occup Ther	3435	6.09	46	Q2	England
Aust Occup Ther J	1998	3.54	44	Q4	Australia
Can J Occup Ther	1895	3.36	53	Q4	Canada
OT Practice	1687	2.99	-	-	-
Occup Ther Health Care	1171	2.08	24	Q3	USA
Scand J Occup Ther	795	1.41	40	Q3	England
Occup Ther Ment Health	758	1.34	20	Q3	England
Occup Ther Now	635	1.13	8	Q4	Canada

^a^ Impact factors according to the Journal Citation Reports (2020 edition) database.

## Data Availability

The data presented in this study are available on request from the corresponding author.

## Supplementary Materials

The following are available online at https://www.mdpi.com/article/10.3390/ijerph182312740/s1, Figure S1: Growth of scientific production over the study period and trend, Table S1: Scientific production of each country by decade and type of study, Table S2: Summary description of the 20 most productive first authors.
