# Supplementary material for: Evolutionary Analysis of International Scientific Output in Occupational Therapy from 1917 to 2020
Source: Int J Environ Res Public Health. 2021 Dec 2;18(23):12740. doi: 10.3390/ijerph182312740 (PMC8656659; doi:10.3390/ijerph182312740)
Supplement: Supplementary file 1 [file ijerph-18-12740-s001.zip › Suplementary table S1.pdf]

CONTINENT				Journa Article	Case Study	Review	Systematic Review	Case Reports	Clinical Trial	Comparative Study	Randomized Controlled Trial	Practice Guideline	Meta-analyses	Controlled Clinical Trial	Evaluation Study	Guideline	Validation Study	Multicenter Study	Clinical Study	Clinical Trial Protocol	Observational Study	Total
AFRICA	1971-1980		*Egypt*	2																		2
			Nigeria	2																		2
			South Africa	13																		13
			Otros	3																		3
		Total		20																		20
	1981-1990		*Algeria*	2																		2
			South Africa	20																		20
			Zimbabwe	3																		3
			Otros	4																		4
		Total		29																		29
	1991-2000		*Egypt*	6																		6
			Nigeria	2																		2
			South Africa	9																		9
			Otros	2																		2
		Total		19																		19
	2001-2010		*Egypt*	8				1														9
			Nigeria	5																		5
			South Africa	67																		67
			Otros	13																		13
		Total		93				1														94
	2011-2020		*Egypt*	21																		21
			Nigeria	16																		16
			South Africa	150		1									2							153
			Otros	42																		42
		Total		229		1									2							232
	Total		*Egypt*	38				1														39
			Nigeria	26																		26
			South Africa	259		1									2							262
			Otros	67																		67
		Total		390		1		1							2							394
	1921-1930		*United States*	99																		99
		Total		99																		99
	1931-1940		*Canada*	24																		24
			United States	110																		110

AMERICA

	Total		134																		134
1941-1950		*Canada*	23																		23
		United States	99																		99
	Total		122																		122
1951-1960		*Canada*	30																		30
		United States	35																		35
	Total		65																		65
1961-1970		*Canada*	21																		21
		United States	94																		94
	Total		115																		115
1971-1980		*Canada*	123																		123
		Mexico	8																		8
		United States	716																		716
		Otros	18																		18
	Total		865																		865
1981-1990		*Canada*	205	1	1			3	1												211
		Mexico	8																		8
		United States	1511		13		24	7	4												1559
		Otros	9																		9
	Total		1733	1	14		24	10	5												1787
1991-2000		*Brazil*	8																		8
		Canada	468	6	20		5	6	1				1								507
		United States	2018		46		54	16	18				3			1	2				2158
		Otros	5																		5
	Total		2499	6	67		59	22	20				4			1	2				2680
2001-2010		*Brazil*	77	1					1					1							80
		Canada	869	5	6	2	2	4	4					3			1				896
		United States	2668	6	12	1	22	7	11	5				3		1	1				2737
		Otros	33				1														34
	Total		3647	12	18	3	25	11	16	5				7		1	2				3747
2011-2020		*Brazil*	531	17	12	19		3		2						1					585
		Canada	1251	8	12	20		2	2	2		1					1				1299
		United States	4418	30	32	19	3	10	2	4	2	1				3				2	4526
		Otros	146			1	1		1												149
	Total		6346	55	57	60	4	15	5	8	2	2				4	1			2	6561
Total		*Argentina*	20			1															21
		Brazil	627	18	12	19		3	1	2				1		1					684
		Canada	3014	20	39	22	7	15	8	2		1	1	3			2				3134
		Chile	42		1		2														45
		Colombia	64			1			1												66
		CostaRica	2																		2
		Cuba	11																		11
		Dominican Re	1																		1

		Ecuador	3																		3
		Grenada	1																		1
		Hawaii			1				1												2
		Jamaica	1																		1
		Mexico	54																		54
		Panama	1																		1
		Peru	5																		5
		Puerto Rico	7																		7
		United States	11768	36	103	20	103	40	35	9	2	1	3	3		5	3			2	12133
		Uruguay	1																		1
		Venezuela	3																		3
	Total		15625	74	156	63	112	58	46	13	2	2	4	7		6	5			2	16175
1951-1960		*Japan*	3																		3
	Total		3																		3
1961-1970		*Israel*	1																		1
		Japan	2																		2
		Malaysia	1																		1
	Total		4																		4
1971-1980		*India*	22																		22
		Japan	33																		33
		Russia	57																		57
		Otros	43																		43
	Total		155																		155
1981-1990		*India*	14																		14
		Israel	29																		29
		Japan	33																		33
		Otros	27																		27
	Total		103																		103
1991-2000		*Israel*	65	3	1		3		1												73
		Japan	40																		40
		Russia	43																		43
		Otros	79																		79
	Total		227	3	1		3		1												235
2001-2010		*China*	164		3			2	1							1					171
		Israel	110	2	3		1									2					118
		Japan	141	1				1	1	1											145
		Otros	284																		284
	Total		699	3	6		1	3	2	1						3					718
2011-2020		*China*	259			1		2													262
		India	229	12				3		1											245
		Japan	406	1			2	3	1	2											415
		Otros	976					1													977
	Total		1876	16		4	3	9	1	6						2					1917

ASIA	Total		*Afghanistan*	1					1													2
			Bahrain	2																		2
			Bangladesh	9																		9
			Brunei Daruss	2																		2
			China	460		3	1		4	1							1					470
			Cyprus	6																		6
			Georgia	1																		1
			India	330	12				3		1											346
			Indonesia	7																		7
			Iran	179							1						1					181
			Iraq	4																		4
			Israel	398	8	4	2	4		1	1						2					420
			Japan	658	2			2	4	2	3											671
			Jordan	28																		28
			Kazakhstan	1																		1
			Korea	1																		1
			Krivorozhye	1																		1
			Kuwait	20																		20
			Lebanon	8																		8
			Malaysia	55			1															56
			Moscow	10																		10
			Myanmar	1																		1
			Nepal	2																		2
			Pakistan	27																		27
			Philippines	14																		14
			Qatar	9				1														10
			Republic of Ko	1																		1
			Russia	202																		202
			Saudi Arabia	58																		58
			Singapore	89																		89
			South Korea	201							1											202
			SriLanka	7																		7
			Taiwan	223													1					224
			Thailand	37																		37
			United Arab E	11																		11
			Uzbekistan	3																		3
			VietNam	1																		1
		Total		3067	22	7	4	7	12	4	7						5					3135
	1921-1931		*Denmark*	1																		1
			Sweden	1																		1
			United Kingdo	2																		2
		Total		4																		4
	1931-1940		*Germany*	1																		1

			United Kingdo	2															2
		Total		3															3
	1941-1950		*Germany*	1															1
			Norway	2															2
			United Kingdo	6															6
			Otros	6															6
		Total		15															15
	1951-1960		*Denmark*	1															1
			Germany	4															4
			United Kingdo	8															8
			Otros	4															4
		Total		17															17
	1961-1970		*Denmark*	5															5
			Germany	7															7
			United Kingdo	25															25
			Otros	5															5
		Total		42															42
	1971-1980		*France*	101															101
			Germany	426															426
			United Kingdo	190															190
			Otros	493															493
		Total		1210															1210
	1981-1990		*France*	138															138
			Germany	258															258
			United Kingdo	181		1		1						1					184
			Otros	356															356
		Total		933		2		1						1					937
	1991-2000		*Germany*	99		1		1	1										102
			Sweden	252	5	3			1										261
			United Kingdo	475	3	2		2	7										489
			Otros	352	2	2		3	1	1			1	1					363
		Total		1178	10	8		6	11	1			1	1					1216
	2001-2010		*Germany*	562		1			2	5	1			1	1				573
			Sweden	406	2	2	2		2	2					1				417
			United Kingdo	1220	8	8	1	1	2		1	1			3				1245
			Otros	1140	3	1			2	10		3		1			1		1161
		Total		3328	13	18	4	1	9	17	2	4		2	5	1	1		3405
	2011-2020		*Germany*	578		2			3		1		1				1	1	587
EUROPE			*Sweden*	577	5	4	5	1		2	4							2	600
			United Kingdo	1229	17	7	13	1	1			2	1				1		1272
			Otros	2536	5	17	20	3	5	4	7		2	1				1	2601
		Total		4920	27	30	38	5	9	6	12	3	4	1			2	4	5061
	Total		*Austria*	184			1		1	1								1	188

			Belgium	171									1								172
			Belgrade	1																	1
			Bosnia and H	6																	6
			Bulgaria	42																	42
			Croatia	39																	39
			Czech Republ	70		1															71
			Denmark	390	1	1	6		2		4										404
			Deutschland	6		2								1							9
			Eslovaquia	1																	1
			Estonia	1																	1
			Finland	167	2	1															170
			France	682		2		1		4											689
			Germany	1936		4		1	6	5	2		1	1	1			1			1	1959
			Greece	77		2															79
			Hungary	62																	62
			Iceland	23						1											24
			Ireland	268	3	4	3		1												279
			Italy	585	1	1		1		1	1	1		1							592
			Latvia	2																	2
			Lithuania	24		1				6											31
			Luxembourg	1																	1
			Malta	9																	9
			Netherlands	631	2	6	3	3	2			2	1	1			1	1				653
			Norway	248					1		1										250
			Poland	222		1	1			1											225
			Portugal	59																	59
			Romania	43																	43
			Serbia	22																	22
			Slovakia	18																	18
			Slovenia	37				1													38
			Spain	444		4	6		2		1	1									458
			Suisse							1											1
			Sweden	1308	12	9	7	1	3	4	4				1						2	1351
			Switzerland	320	1	1	1		1				1								325
			Turkey	164																	164
			Ukraine	40																	40
			United Kingdo	3338	28	18	14	5	10		1	3	1	1	3			1				3423
			Yugoslavia	9																	9
		Total		11650	50	58	42	13	29	24	14	7	5	5	5		1	3			4	11910
	1941-1950		*Australia*	2																	2
		Total		2																	2
	1951-1960		*Australia*	3																	3
		Total		3																	3

OCEANIA	1961-1970		*Australia*	15																	15
		Total		15																	15
	1971-1980		*Australia*	44																	44
			Kiribati	1																	1
			New Zealand	11																	11
			PapuaNewGu	1																	1
		Total		57																	57
	1981-1990		*Australia*	90	2																92
			New Zealand	6																	6
			Papua New G	1																	1
		Total		97	2																99
	1991-2000		*Australia*	348	4	12		1	1	1											367
			New Zealand	30		2															32
		Total		378	4	14		1	1	1											399
	2001-2010		*Australia*	918	8	14	6	2	2	4			3				1				958
			New Zealand	88	1	1			1												91
			Papua New G	2																	2
		Total		1008	9	15	6	2	3	4			3				1				1051
	2011-2020		*Australia*	1572	14	12	31	2	2		5		5		1		2	1		1	1648
			New Zealand	100	1	4	2	1					1								109
			Solomon Islar	1																	1
		Total		1673	15	16	33	3	2		5		6		1		2	1		1	1758
	Total		*Australia*	2992	28	38	37	5	5	5	5		8		1		3	1		1	3129
			New Zealand	235	2	7	2	1	1				1								249
			Papua New G	4																	4
			Otros	2																	2
		Total		3233	30	45	39	6	6	5	5		9		1		3	1		1	3384
