# Supplementary material for: Evolutionary Analysis of International Scientific Output in Occupational Therapy from 1917 to 2020
Source: Int J Environ Res Public Health. 2021 Dec 2;18(23):12740. doi: 10.3390/ijerph182312740 (PMC8656659; doi:10.3390/ijerph182312740)
Supplement: Supplementary file 1 [file ijerph-18-12740-s001.zip › Supplementary table S2.pdf]

Author	Affiliation	h-index	Country	Documents as first author	Total documents	% First author
**Strzelecki, M V**		1		70	71	98.59%
**Brown, T**	Monash university /faculty of medicine/ associate editor/ school of primary and allied health care	26	Australia	66	126	52.38%
**Kielhofner, G**	University of illinois at chicago	33	United states	59	132	44.70%
**Gutman, S A**	Columbia university/ rutgers university-newark campus	14	United states	59	74	79.73%
**Lloyd, C**	Unsw sydney / black dog institute	23	Australia	58	92	63.04%
**Waite, Andrew**				58	58	100%
**Rogers, J C**	University of pittsburgh	35	United states	54	64	84.38%
**Eklund, M**	Lunds universitet/ institutionen for halsovetenskaper/ malmo hogskola	36	Sweden/ denmark	52	119	43.70%
**Johnson, J A**	Occupational therapy in health care	0	United states	49	51	96.08%
**Neistadt, M E**	University of new hampshire durham	14	United states	46	46	100%
**Case-Smith, J**	The ohio state university	28	United states	44	58	75.86%
**Peloquin, S M**	Ut medical branch at galveston	15	United states	43	47	91.49%
**Rodger, S**	The university of queensland / cooperative research centre for living with autism crc	34	Australia	41	110	37.27%
**Yerxa, E J**	University of southern california	18	United states	41	48	85.42%
**Llorens, L A**	San jose state university	7	United states	39	45	86.67%
**Hasselkus, B R**	University of wisconsin-madison	20	United states	38	48	79.17%
**Law, M**	Mcmaster university/ canchild centre for childhood disability research	62	Canada	36	103	34.95%
**Cusick, A**	The university of sydney/ western sydney university	23	Australia	34	68	50%
**Oliveck, M**				34	34	100%
**Hinojosa, J**	Nyu steinhardt	16	United states	33	67	49.25%

Table S2. The top 20 most productive first author
