# Supplementary figures and images for: Evolutionary Analysis of International Scientific Output in Occupational Therapy from 1917 to 2020
Source: Int J Environ Res Public Health. 2021 Dec 2;18(23):12740. doi: 10.3390/ijerph182312740 (PMC8656659; doi:10.3390/ijerph182312740)

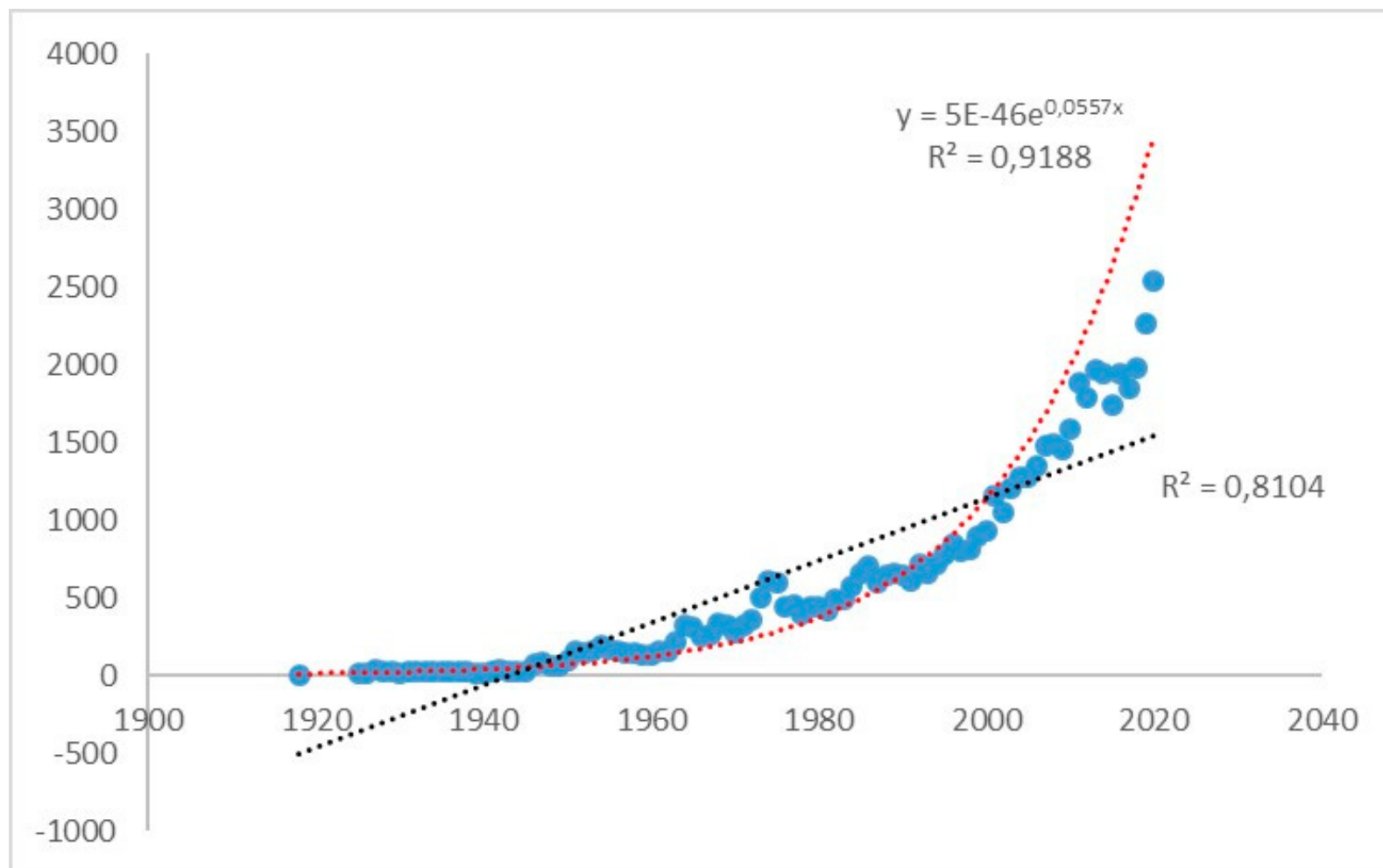

Supplement: Supplementary file 1 [file ijerph-18-12740-s001.zip › Supplementary FigureS1.pdf]
